# Ceftazidime–Avibactam versus Meropenem for the Treatment of Complicated Intra-Abdominal Infections

**DOI:** 10.3390/antibiotics8040255

**Published:** 2019-12-06

**Authors:** Che-Kim Tan, Chih-Cheng Lai, Chien-Ming Chao

**Affiliations:** 1 Department of Intensive Care Medicine, Chi Mei Medical Center, Yongkang, Tainan 710, Taiwan; chekim.tan@gmail.com; 2 Department of Internal Medicine, Kaohsiung Veterans General Hospital, Tainan Branch, Tainan 813, Taiwan; dtmed141@gmail.com; 3 Department of Intensive Care Medicine, Chi Mei Medical Center, Liouying, Tainan 710, Taiwan

**Keywords:** ceftazidime–avibactam, meropenem, complicated intra-abdominal infection

## Abstract

This study reports an integrated analysis of three randomized controlled trials to compare the clinical efficacies and safety of the ceftazidime–avibactam (CAZ–AVI) combination and meropenem in the treatment of adult patients with complicated intra-abdominal infections (cIAIs). Overall, a total of 1677 patients (CAZ–AVI: 835 patients; meropenem: 842 patients) were included in this analysis. CAZ–AVI had a clinical cure rate at test of cure in the clinically evaluable (CE) population similar to that of meropenem (OR, 0.88; 95% CI, 0.58–1.32; *I*^2^ = 0%). Similar trends were also observed in the modified intent-to-treat (MITT) population (OR, 0.80; 95% CI, 0.59–1.09; *I*^2^ = 0%) and microbiological evaluable (ME) population (OR, 0.73; 95% CI, 0.32–1.68; *I*^2^ = 0%). In terms of clinical cure rate at the end of treatment, the efficacy of CAZ–AVI was comparable to that of meropenem in the CE population (OR, 0.77; 95% CI, 0.47–1.25; *I*^2^ = 0%), MITT population (OR, 0.70; 95% CI, 0.47–1.06; *I*^2^ = 5%), and ME population (OR, 1.26; 95% CI, 0.39–4.08; *I*^2^ = 0%). CAZ–AVI had a similar risk of (i) treatment emergent adverse events (TEAEs) (OR, 1.03; 95% CI, 0.79–1.36; *I*^2^ = 38%), (ii) any serious adverse events (OR, 0.97; 95% CI, 0.67–1.40; *I*^2^ = 0%), (iii) discontinuation of study drug due to TEAE (OR, 2.14; 95% CI, 1.00–4.57), and iv) all-cause mortality (OR, 1.66; 95% CI, 0.78–3.53; *I*^2^ = 0%) when compared with meropenem. In conclusion, CAZ–AVI had comparable efficacy and safety profile to those of meropenem in the treatment of cIAI.

## 1. Introduction

Intra-abdominal infection is a serious type of infection, which can cause high morbidity and mortality. In addition to source control by radiological or surgical intervention, appropriate antibiotic therapy is essential in the management of complicated intra-abdominal infections (cIAIs) [1]. Carbapenem exhibits broad-spectrum activity and is commonly prescribed for treating cIAI. However, several surveillance investigations have shown the emergence of carbapenem resistance among the pathogens causing the clinical condition of cIAI [2,3]. Thus, a new antibiotic is urgently needed in the management of multi-drug resistant organism causing cIAI.

Ceftazidime–avibactam (CAZ–AVI) is a newly developed antibiotic combination of a ß-lactam and a ß-lactamase inhibitor [4]. CAV–AVI and carbapenems share some similar pharmacokinetic and pharmacodynamic profiles. Both exhibit time-dependent antimicrobial activity, are administered every 8 h, and their dosage requires adjustment according to the renal function. However, the well-known drug–drug interaction between carbapenem and antiepileptics was not observed for CAV–AVI, making it a better choice in patient with seizures. CAV–AVI exhibited potent in vitro activity against many commonly encountered bacteria, including multi-drug resistant organisms, in several global surveillance investigations [5,6,7,8,9,10]. Clinically, the usefulness of CAZ–AVI has been demonstrated to be comparable to that of carbapenem in the treatment of complicated urinary tract infections (cUTIs) in three randomized controlled trials (RCTs) [11,12,13] and one meta-analysis [14]. In addition to cUTI, there were three more recent RCTs that also compared the effects of CAZ–AVI and carbapenem in the treatment of cIAIs [15,16,17]. To confirm the usefulness of CAZ–AVI in the treatment of cIAI, we conducted an integrated analysis of three recent RCTs [15,16,17], comparing the clinical efficacy and safety of CAZ–AVI with those of meropenem in the treatment of adult patients with cIAI.

## 2. Methods

All three RCTs [15,16,17] were multicenter studies and included hospitalized adult patients with cIAI. Two [16,17] were phase 3 trials, and one [15] was a phase 2 trial. Table 1 and Table 2 summarize the characteristics of the study and the patients. All RCTs [15,16,17] compared CAZ–AVI plus metronidazole versus meropenem. Overall, a total of 1677 patients (CAZ–AVI: 835 patients; meropenem: 842 patients) were included in this analysis. Study populations were defined as (i) clinically evaluable (CE) population, including patients who received the study drug, complied with the protocol, and had a clinical response assessed at the test-of-cure visit (TOC), (ii) modified intent-to-treat (MITT) population, including all intent-to-treat patients who received at least one dose of the study drug, (iii), microbiological MITT (mMITT) population, comprising MITT patients who met the disease definition of cIAI and had a baseline pathogen, (iv) microbiological evaluable (ME) population, including CE patients who had an identified baseline pathogen and whose microbiological response was assessed. The primary outcome was clinical cure rate at the TOC, two weeks after the last dose of the study drug [15] or 28–35 days after randomization. [16,17] Clinical cure was defined as resolution of all or most pretherapy signs or symptoms, with no further requirement for antibiotics, radiological intervention, or surgery. Secondary outcomes included clinical cure rate at the end of treatment (EOT) and the risk of adverse events.

## 3. Results

**Figure 1:**
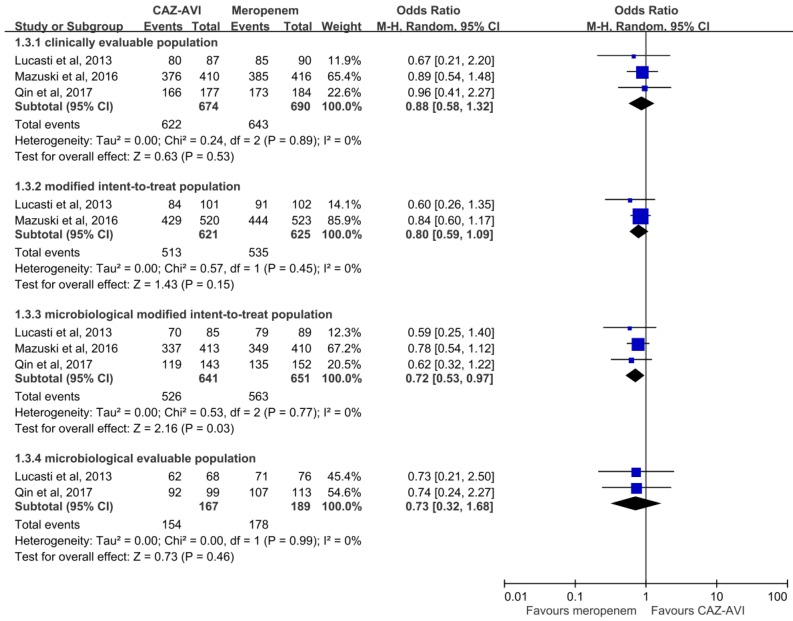
Clinical cure rates at test of cure visit of ceftazidime–avibactam (CAZ–AVI) and meropenem.

Overall, CAZ–AVI had a clinical cure rate at TOC in the CE population similar to that of meropenem (622 (92.3%) vs. 643 (93.2%), OR, 0.88; 95% CI, 0.58–1.32; *I*^2^ = 0%, Figure 1) in the pooled analysis of the three RCTs [15,16,17]. Similar trends were also observed in the MITT population (513 (82.6%) vs. 535 (85.6%), OR, 0.80; 95% CI, 0.59–1.09; *I*^2^ = 0%) and ME population (154 (92.2%) vs. 178 (94.25), OR, 0.73; 95% CI, 0.32–1.68; *I*^2^ = 0%) in the pooled analysis of two studies. However, the pooled analysis of the mMITT population showed CAZ–AVI was associated with a lower clinical cure rate when compared with meropenem (526 (82.1%) vs. 535 (85.6%), OR, 0.72; 95% CI, 0.53–1.97; *I*^2^ = 0%). In terms of clinical cure rate at EOT, the efficacy of CAZ–AVI was comparable to that of meropenem in the CE population (648 (94.3%) vs. 670 (95.6%), OR, 0.77; 95% CI, 0.47–1.25; *I*^2^ = 0%), MITT population (552 (88.9%) vs. 575 (92.0%), OR, 0.70; 95% CI, 0.47–1.06; *I*^2^ = 5%) and ME population (169 (97.1%) vs. 187 (96.4%). OR, 1.26; 95% CI, 0.39–4.08; *I*^2^ = 0%), but lower than that of meropenem in the mMITT population (487 (87.6%) vs. 519 (92.3%), OR, 0.59; 95% CI, 0.39–0.87; *I*^2^ = 0%).

In terms of safety, CAZ–AVI had a similar risk of (i) treatment emergent adverse events (TEAEs) (390 (46.1%) vs. 369 (43.5%), OR, 1.11; 95% CI, 0.92–1.35; *I*^2^ = 0%), (ii) any serious adverse events (60 (7.1%) vs. 62 (7.3%), OR, 0.97; 95% CI, 0.67–1.40; *I*^2^ = 0%), (iii) discontinuation of study drug due to TEAE (21 (2.8%) vs. 10 (1.3%), OR, 2.14; 95% CI, 1.00–4.57), and (iv) all-cause mortality (18 (2.1%) vs. 11 (1.3%), OR, 1.66; 95% CI, 0.78–3.53; *I*^2^ = 0%) when compared with meropenem.

For common adverse event, CAZ–AVI had a higher risk of nausea (64 (7.6%) vs. 34 (4.0%), OR, 2.10; 95% CI, 1.09–4.03; *I*^2^ = 42%) and vomiting (43 (5.1%) vs 19 (2.2%), OR, 2.34; 95% CI, 1.34–4.08; *I*^2^ = 0%) but had the similar risk of pyrexia (42 (5.0%) vs. 48 (5.7%), OR, 0.87; 95% CI, 0.57–1.34; *I*^2^ = 0%) and cough (20 (2.7%) vs. 25 (2.9%), OR, 0.81; 95% CI, 0.41–1.58; *I*^2^ = 12%) when compared with meropenem.

## 4. Discussion

In this study, we demonstrated that the clinical efficacy of CAZ–AVI was comparable to that of meropenem on the basis of the integrated analysis of three RCTs [15,16,17]. This evidence was supported by an analysis based on different populations (CE, MITT, and ME) and different outcome measurements (clinical cure rate at TOC and EOT). The only exception was the finding regarding of the mMITT population, for whom the clinical cure rate of CAZ–AVI was lower than that of meropenem. Most of our findings are consistent with those of Chen et al.’s meta-analysis [14], in which there were no significant differences between CAZ–AVI and carbapenems in clinical success and microbiological success for the treatment of Enterobacteriaceae infections. However, in contrast to Chen et al.’s analysis [14], which included two RCTs of cUTIs, this study focused only on cIAI and thus helps expand the application of CAZ–AVI. In addition, our findings are in line with those of Sternbach et al.’s meta-analysis [18], in which CAZ–AVI was comparable in efficacy to meropenem and other antibiotics for treating cIAIs. In contrast, our study compared CAZ–AVI only with meropenem. In summary, the present study results indicate that CAZ–AVI is comparable in efficacy to meropenem in the treatment of cIAIs. Therefore, CAZ–AVI could be a therapeutic alternative for treating cIAIs according to the findings of our study and of previous reports [14,18].

In addition to clinical efficacy, the risk of adverse events associated with CAZ–AVI administration is an important concern. Our analysis showed that gastrointestinal adverse events were the most common adverse events in patients treated with CAV–AVI, and the risk of nausea and vomiting was higher in the CAV–AVI group than in the meropenem group. However, the risk of adverse events for CAZ–AVI was similar to that of meropenem in relation to TEAEs, serious adverse events, discontinuation of study drug due to TEAE, and all-cause mortality. Overall, this suggests that CAZ–AVI is as tolerable as meropenem in the treatment of cIAI.

This study has several limitations. First, because only three RCTs investigated this issue, the number of patients was limited in this study. Second, we did not evaluate the clinical response to CAZ–AVI in patients with cIAI caused by individual pathogens, especially multidrug-resistant organisms. Third, the definition of TOC varied in the three included studies [15,16,17], which may affect the results. Therefore, we cannot investigate the cause of the lower clinical cure rate of CAZ–AVI in the mMITT population. Further study is warranted to clarify these issues.

In conclusion, CAZ–AVI had comparable efficacy to that of meropenem in the treatment of cIAI. In addition, CAZ–AVI was as tolerable as meropenem in this analysis. Therefore, CAZ–AVI could be a therapeutic option in the treatment of cIAIs.

## Figures and Tables

**Table 1:** Characteristics of the included studies. cIAI: complicated intra-abdominal infections.

Study	Study Design	Study Period	Study Population	Study Group (No. of Patients)	Control Group (No. of Patients)
Lucasti et al., 2013	Multicenter, randomized, active-controlled, double-blind trial	2009	cIAI in hospitalized adults	ceftazidime/avibactam plus metronidazole (101)	Meropenem (102)
Mazuski et al., 2016	prospective, randomized, multicenter, double-dummy, double-blind, comparative, global studies	2012–2014	cIAI in hospitalized adults	ceftazidime/avibactam plus metronidazole (520)	Meropenem (523)
Qin et al., 2017	multicenter, randomized, double blind, double-dummy, comparative study	2013–2015	cIAI in hospitalized adults	ceftazidime/avibactam plus metronidazole (214)	Meropenem (217)

**Table 2:** Baseline characteristics of the patients.

Study	Age, Mean (SD)		Male, No. (%)		Predominant Race, No. (%)		APACHE II Score ≤ 10, No. (%)		Monomicrobial Infection, No. (%)	
	CAV-AVI	MEP	CAV-AVI	MEP	CAV-AVI	MEP	CAV-AVI	MEP	CAV-AVI	MEP
Lucasti et al., 2013	43.0 (15.9)	42.6 (18.1)	70 (69.3)	81 (79.4)	White: 56 (55.4)	White: 65 (63.7)	84 (83.2)	85 (83.3)	NR	NR
Mazuski et al., 2016	49.8 (17.5)	50.3 (18.3)	326 (62.7)	332 (63.5)	White: 403 (77.5)	White: 396 (75.7)	437 (84.0)	434 (83.0)	209 (40.2)	205 (39.2)
Qin et al., 2017	48.5 (16.8)	48.5 (17.4)	141 (65.9)	153 (70.5)	Chinese: 127 (59.3)	Chinese: 135 (62.2)	201 (93.9)	201 (92.6)	84 (39.3)	101 (46.5)

NR: not reported, MEP: meropenem.
